# Debate: Where to next for universal school‐based mental health interventions? Universal versus targeted school‐based mental health interventions: a health economic perspective

**DOI:** 10.1111/camh.12751

**Published:** 2024-12-10

**Authors:** Paul McCrone

**Affiliations:** ^1^ Institute for Lifecourse Development University of Greenwich London UK

**Keywords:** Universal interventions, cost‐effectiveness, school‐based, health economics

## Abstract

Mental health problems in schools are a concern and various initiatives have been developed to address these. Interventions can be universal (covering a whole school) or targeted (addressing those with specific needs). Any new intervention should be evaluated, and this should include an assessment of cost‐effectiveness. Evidence to date suggests that while gains from universal schemes may be modest, they can still be cost‐effective given the extent of their reach. However, targeted interventions can address key health inequalities which should also be an objective of an economic evaluation. Studies that have examined the longer term impact of both universal and targeted approaches have demonstrated that both can represent good values for money, and it is likely that a blended or tiered approach is appropriate.

## Introduction

All types of health and social care system require decisions to be made about who to support and how best that can be done. Within healthcare, there will almost always be alternative ways of providing care and evaluations of these alternatives can inform the decision‐making process. In the absence of evaluations, policymakers may decide to continue with the usual way of doing things or may opt for alternatives on the basis of limited evidence. With alternative ways of addressing a particular clinical or social issue, such as occurrence of mental health problems in young people, we of course have to assess efficacy and effectiveness. Given scarce resources, particularly professional inputs, we also have to establish which approaches represent the best value for money. The conventional way in which this is achieved in many countries is to use the methods of economic evaluation where the extra costs incurred to achieve improvements in outcomes are determined and a value judgement is made regarding these figures. If an intervention results in lower costs and better outcomes than an alternative, then it is ‘dominant’. However, we are frequently faced with situations where costs are higher and outcomes better than the alternative.

In many countries, the outcome measure used by decision‐makers is the quality‐adjusted life year (QALY) and the value judgement is formalised in a threshold. In England and Wales, the threshold is £20,000 per QALY—interventions achieving a figure below this are likely to be approved by the regulatory body. While much of this is of a technical nature, judging cost‐effectiveness ratios against a threshold is built on a utilitarian premise whereby we wish to maximise population health. What it does not do is to explicitly allow us to prioritise interventions, and hence spending, on those with the greatest level of need. That may happen but this could be by coincidence.

This is all very pertinent to the issue of whether we should invest in universal school‐based programmes or those that are more targeted. A universal programme may result in modest incremental costs per pupil compared to the status quo and while it may result in benefits, these could be small and still achieve large improvements in aggregated pupil mental health and well‐being. A targeted approach could be more expensive per pupil receiving it and gains would be aggregated across a smaller number. As such, the universal approach could be shown to be more cost‐effective while only delivering modest improvements.

## What is already known?

The MYRIAD study (Kuyken et al., 2022) compared a mindfulness‐focused resilience training package provided to schools across the United Kingdom with treatment as usual (standard emotional training). The primary outcomes (risk of depression, emotional functioning and well‐being) were not significantly improved as a result of the intervention and so this approach was not favoured. However, the economic evaluation conducted as part of the trial showed that intervention had an 83% likelihood of being cost‐effective. This was due to the intervention only having slightly higher costs (by £6.84) per pupil than treatment as usual, which when divided by the extra QALYs gained (0.012 on average) resulted in a very favourable incremental cost‐effectiveness ratio. Given this, if policymakers had to choose between one of these two options, and if cost‐effectiveness were the only consideration, then they should opt for the mindfulness intervention. However, that assumes that these are the only options, which is not the case as other forms of universal intervention may be possible in addition to targeted approaches.

An economic evaluation that did compare universal and targeted interventions was conducted by Lee et al. (2023). This multi‐country study compared the cost‐effectiveness of both approaches in terms of reducing the incidence of depression/anxiety and deaths from suicide. Given that these were essentially public health interventions, both costs and outcomes (healthy life years gained) were reported at population levels. The findings revealed that the cost (in international dollars which account for cost of living differences) per healthy life year gained in lower income countries was I$958 for universal interventions and I$11,123 for targeted approaches. In higher income countries, the figures were I$2006 and I$18,473, respectively. If our guiding principle is one of economic efficiency then, given limited resources, this study would indicate investing in universal rather than targeted approaches. However, as the authors rightly point out, we also need to consider health inequalities and as such provision of targeted support when needed should be available.

A recent overview of existing systematic reviews of evaluations of targeted and universal interventions has been conducted (Zbukvic et al., 2024). Fourteen reviews were identified, and it was shown that most interventions used some form of cognitive behavioural therapy (CBT). The evidence pointed to targeted interventions having short‐term effectiveness in addressing depression and anxiety, but with limited long‐term effects. Evidence on universal interventions was more mixed, but again short‐term gains from a CBT‐focussed approach seemed to be effective. The evidence to support resilience focussed interventions was far more limited. This review did not explicitly address economic issues.

While some interventions have aimed at improving well‐being or strengthening resilience, with as the hope of a reduction in depression and anxiety, others have focussed more on specific issues such as substance abuse or problem drinking. In one study, a cost–benefit analysis was conducted alongside a clustered randomised trial of a preventative approach focussed on peer support in schools in the northwest of the USA (Van Ryzin, Cil, & Roseth, 2022). The findings showed significant reductions in substance abuse in intervention schools, and for every dollar invested in the intervention, there was a return of up to $9–$101. Elsewhere, Teesson et al found some evidence that a universal, digital, school‐based approach has some benefits over 30 months in terms of increasing knowledge about mental health problems and reducing the likelihood of some adverse drinking behaviours and anxiety symptoms (Teesson et al., 2020).

## Methodological issues

Evidence is clearly mixed but there are indications that while universal interventions can produce modest benefits, targeted approaches seem to be reasonably effective in addressing emergent mental health problems. There are though limitations (which relate to each other) in the methods, we commonly use to assess both effectiveness and cost‐effectiveness. First, trials, while crucial, produce average effects while these may be positive there are likely to be some who do worse following an intervention. There have been concerns that universal school‐based interventions may cause harm for some and when the target population is substantial, this can be a major worry (Foulkes, Andrews, Reardon, Reardon, & Stringaris, 2024). Second, the consequence of trials and other comparative studies is that the ‘winner takes all’. While a study may well show that one approach is on average better than another and possibly more cost‐effective, the reality is that the alternative is likely to be appropriate in some circumstances. Blended approaches are probably more appropriate—providing universal support to address general issues coupled with targeted interventions for those with specific identified needs. Third, most economic evaluations are designed to demonstrate which of two or more options for addressing a specific health or societal need are the most efficient (i.e. which achieves specific outcomes at the lowest cost). Few studies are explicitly set up to show which approaches are most likely to lead to reductions in health inequalities. Fourth, few studies consider all available alternatives. As such, far more attention needs to be given to synthesising results from individual evaluations using models and incorporating evidence that has already been produced.

## Conclusions

Relatively few economic evaluations of either universal or targeted approaches for providing mental health support in schools have been conducted. Nevertheless, we can still draw some tentative conclusions based on the broader effectiveness data and the few economic studies that do exist. When we consider the cost per pupil, universal interventions are likely to be relatively inexpensive. As such, even modest improvements in well‐being, mental health and quality of life can result in favourable cost‐effectiveness ratios as shown with the MYRIAD study (Kuyken et al., 2022). Such a finding could apply to many other public health interventions. Therefore, given current ways of allocating funds such an investment seems reasonable. However, to address the inequalities arising through some children and adolescents having, or being at risk of developing, more serious problems a tiered or blended approach with a combination of interventions included targeted ones may be more appropriate. Economic evaluations of such blended approaches should be conducted.

What is apparent is that the approaches we use to derive economic evidence for school‐based mental health interventions could be improved. Our general methods that are used may not be easily applied when the effects of interventions could be disparate and occurring over a prolonged time horizon. A benefit of both universal and targeted interventions may simply not be apparent unless we make extrapolations into the future, and the studies that have done this (e.g.Lee et al., 2023; Van Ryzin et al., 2022) have demonstrated that universal and targeted programmes can indeed represent good value for money (Lee et al., 2023; Van Ryzin et al., 2022). There is sufficient evidence to suggest that both have a place.

## Conflict of interest statement

The author has declared that they have no competing or potential conflicts of interest.

## Funding information

None.

## Ethical information

No ethical approval was required for this article.

## Data Availability

Data sharing not applicable to this article as no datasets were generated or analysed during the current study.
